# Experimental Measurement-Device-Independent Entanglement Detection

**DOI:** 10.1038/srep08048

**Published:** 2015-02-04

**Authors:** Mohamed Nawareg, Sadiq Muhammad, Elias Amselem, Mohamed Bourennane

**Affiliations:** 1 Physics department, Stockholm University, S-10691, Stockholm, Sweden; 2 Instytut Fizyki Teoretycznej i Astrofizyki, Uniwersytet Gdański, PL-80-952 Gdańsk, Poland

## Abstract

Entanglement is one of the most puzzling features of quantum theory and of great importance for the new field of quantum information. The determination whether a given state is entangled or not is one of the most challenging open problems of the field. Here we report on the experimental demonstration of measurement-device-independent (MDI) entanglement detection using witness method for general two qubits photon polarization systems. In the MDI settings, there is no requirement to assume perfect implementations or neither to trust the measurement devices. This experimental demonstration can be generalized for the investigation of properties of quantum systems and for the realization of cryptography and communication protocols.

Quantum entanglement leads to the most counterintuitive effects in quantum mechanics^1^,^2^ and plays a central role in the field of quantum information, leading to ongoing efforts for its quantitative and qualitative characterization. While entanglement of pure bipartite states is well understood^3^,^4^, the entanglement of mixed and multipartite systems is still under intense research^5^. Studies of entanglement are particularly relevant for evaluating its use as a resource for quantum communication and quantum computation^6^. Protocols like quantum teleportation and super dense coding can not be performed without shared entanglement between the communicating parties.

A simple and practical tool for the entanglement detection is based on the concept of entanglement witness (EW). A witness operator that detects entanglement of a pure non-separable state |*ψ*〉 is given by


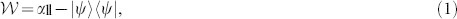
where 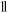 is the identity operator, *α* = max_|*ϕ*〉∈*S*_|〈*ϕ*|*ψ*〉|^2^, and *S* denotes the set of separable states. This method guarantees that 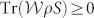 for all separable states *ρS*, and that 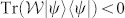. Thus, a negative expectation value of the observable 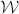 clearly signifies that the state |*ψ*〉 is entangled^7^. For the experimental and practical implementation of this witness method, the witness operator have to be optimally decomposed into a minimal number *K* of *local* von Neumann (or projective) measurements *M_k_* with *k* ∈ {1, …, *K*}^8^,^9^. This method was experimentally implemented for the entanglement detection of a two-qubit system^10^ and for multipartite system^11^.

A disadvantage of this entanglement detection technique using EWs is that it requires a perfect implementation of measurement devices. An imperfect measurement can lead to an incorrect estimation of 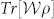, and possibly the wrong entanglement detection^12^,^13^,^14^. An alternative way to avoid this problem is to rely on the loophole-free violation of a Bell inequality^2^. In these conditions the violation guarantees the presence of entanglement, independently of the types of measurement performed, the precision involved in their implementation, or on assumptions about the relevant Hilbert space dimension^15^. However, even for two-qubit systems there exist entangled states which do not violate any Bell inequality^16^.

Very recently, a new entanglement detection method has been proposed, the so-called measurement-device-independent entanglement witnesses (MDI-EWs) where the entanglement verification do not depend on the particular functioning of the measuring devices^17^. This method of MDI-EW was based on and related to the so-called nonlocal games with quantum inputs, proposed by Buscemi^18^. In such games, two users Alice and Bob located at distant locations, like to persuade a referee that they share an entanglement resource. This situation can correspond to the case where the referee does not have direct access to the resource or he does not trust the users. It is insufficient for the users to transmit a list of their local measurements settings and their corresponding outcomes to the referee^19^, even if with these given two lists he is able to violate Bell inequality. For example Gerhardt *et al* have experimentally faked the violation of Bell inequality with classical light by controlling Alice's and Bob's detectors^20^.

To ensure that the users cannot conspire to mimic entanglement, Buscemi has shown that if the referee sent two quantum states, *τ_s_* to Alice and *ω_t_* to Bob and request two output values a and b from Alice and Bob respectively (see Fig. 1) then an entanglement detection can be obtained by the referee. More precisely, in this scenario, Alice and Bob do share some quantum state *ρ_AB_*. They can communicate before to agree on a pre-established strategy but they are not allowed to communicate after the referee has sent them the states *τ_s_* and *ω_t_*. The values *a* and *b* are the success/failure of the joint projections of the referee states *τ_s_* and *ω_t_* and the local part of the shared state *ρ_AB_* on to a maximally entangled state for Alice and Bob respectively. The correlation between values a and b is characterized by the conditional probability distribution *P*(*a*, *b*|*τ_s_*, *ω_t_*).

It has been shown that if their state is entangled, then the referee can obtain, by asking Alice and Bob to perform joint measurements on their respective part of *ρ_AB_* and on the input quantum states sent by him, a linear combination *I* of probabilities *P*(*a*, *b*|*τ_s_*, *ω_t_*)


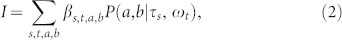
where *β_s_*_,*t*,*a*,*b*_ are real numbers, which cannot be explained without entanglement^17^,^18^.

With the proper optimization of *β_s_*_,*t*,*a*,*b*_ The referee obtains *I* < 0 for the entangled states and *I* ≥ 0 necessarily hold for all separable states shared between Alice and Bob. We like to note that the MDI method is also proposed in the context of quantum key distribution (QKD) between two partners Alice and Bob where the state preparations of Alice and Bob are trusted, but not their measurement devices^21^.

It has been proven that any standard EW can be used to derive an explicit form for MDI-EW with quantum inputs^17^. The requirement that with standard EWs the measurement implementations must be trusted is replaced by joint measurement with input quantum states.

Consider a bipartite qubit entangled state *ρ_AB_*. Let 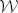 be an EW detecting the entanglement of *ρ_AB_*, i.e., a Hermitian operator such that 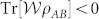, while 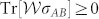 for all separable states *σ_AB_*. 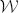 can be written in the form


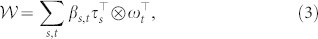
where *β_s_*_,*t*_ are real coefficients and the operators 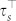 and 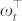 are density matrices (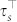 and 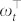 are the transposes of *τ_s_* and *ω_t_* respectively).

We use the decomposition (3) to obtain a MDI-EW as follows: Alice and Bob receive input states *τ_s_* and *ω_t_* respectively from the referee. Then they project their part of shared state together with these input states onto a maximally entangled state. If the projection is successful, they outcome a result 1 otherwise they outcome the result 0.

The expression
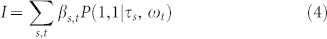
is then of the form (2) [with *β_s_*_,*t*,1,1_ = *β_s_*_,*t*_ and *β_s_*_,*t*,*a*,*b*_ = 0 for (*a*, *b*) ≠ (1, 1)], and takes non-negative values *I* ≥ 0 when Alice and Bob do not share entanglement, while sharing an entangled state *ρ_AB_* allows them to get a correlation *P* such that *I* < 0.

For the experimental MDI-EW, let's consider the 2-qubit Werner state^16^


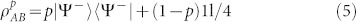
where *p* ∈ [0, 1], 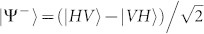 is the singlet state, and 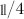 is the maximally mixed state. The physical qubits are single polarized photons and the computational basis corresponds to horizontal *H* and vertical *V* linear polarization |0〉 ≡ |*H*〉 and |1〉 ≡ |*V*〉. 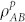 is entangled if and only if *p* > 1/3, and can be detected with the EW


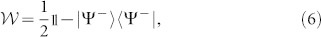
such that 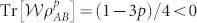 for *p* > 1/3, while 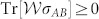 for all separable 2-qubit states *σ_AB_*.

To explicitly express MDI-EW in the form of Eq. (4), i.e, to obtain the *β_s_*_,*t*_ coefficients, one has to decompose 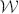 of Eq. (6) in the form of Eq. (3). Then we get


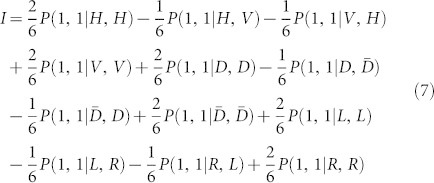
where the six input quantum states |*H*〉, |*V*〉, |*D*〉, 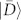, |*L*〉, and |*R*〉 are sent by the referee. Importantly, it has been shown that MDI-EW is loss tolerant including loss due to the transmission and low quantum efficiency of the detectors^17^.

The experimentally prepared Werner state is a two-qubit polarization mixed entangled state with a mixture of the singlet two-qubit |*ψ*^−^〉 and the maximally mixed 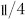 state. To achieve this photon pair emitted in two spatial modes (A,B) through the process of spontaneous parametric downconversion (SPDC)^22^ are used (Fig. 2) together with randomized bitflip and phaseflip operations.

We use UV femtosecond pulses of a frequency-doubled mode-locked Ti:sapphire laser to pump a 2 mm *β*-barium borate (BBO) crystal at a wavelength of 390 nm with an average pump power of 650 mW. First we prepare maximally polarization entangled state in singlet form 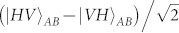. Next we transform randomly the singlet |*ψ*^−^〉 to any of the others Bell states |*ψ*^+^〉, |*ϕ*^−^〉, and |*ϕ*^+^〉. These operators were experimentally realized by motorized rotating four quarter wave plates QWP1, QWP2,QWP3, and QWP4 placed in modes (A) and (B)(see table 1 for the QWP settings). These motors are controlled by a computer that randomly configures the settings according to the desired weight *p* (Fig. 2).

Alice and Bob six quantum input states are single photon polarization states 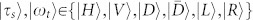. These photons are generated from a second and a third SPDC process and emitted in the arms C and D (see Fig. 2). The preparation setups for these states consist of combinations of polarizer POLA, half wave plate HWPA, and quarter wave plate QWPA for Alice and a similar setup for Bob, POLB, HWPB, and QWPB (see table 2 for the Alice and Bob settings). To exactly define the spatial and spectral properties of the emitted four photons, they are coupled into single mode fibers (SMF) and passed through narrow band interference filters (F) placed in modes A, B, C, and D.

The joint measurement at Alice and Bob sides are Bell measurement (analyzers) on their respective qubit and the qubit sent by the referee^23^. The experimental setup for Bell analyzers consist of polarization beam splitter (PBS) and half wave plates (HWP) oriented at 22.5°. All measurements in the two output modes of Alice's and Bob's analyzers are performed with polarization analysis components. The photons are detected by Si avalanche photodiodes *D_i_*(*i* = 1, …8, *T*_1_, *T*_2_), and the coincidences are registered with a ten channels (one counting channel for each of Alice's and Bob's measurement detectors and two for the Referee's trigger detectors) multi-coincidence logic unit. This two-qubit Bell analyzers at Alice and Bob consists of coherent interference of the modes A and C for Alice and B and D for Bob at a polarization beam splitter (PBS). To obtain indistinguishability of the photons A and C and of the photons B and D due to their arrival times we adjusted the path length of the photon in mode C and in mode D, respectively using delay lines. In Fig. 3, the four-fold coincidence between the detectors versus the delay path between the photons A and C and B and D are shown. Sub-figures (a) and (c) show the four-fold coincidence for |*V*〉 and |*H*〉 input quantum states in mode C at Alice, singlet 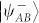 in modes A and B, and the trigger photon in mode *T*_1_ at the referee. Sub-figures (b) and (d) show the four-fold coincidence for |*V*〉 and |*H*〉 input quantum states in mode D at Bob, singlet 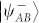 in modes A and B, and the trigger photon in mode *T*_2_ at the referee. The average four-photon coincidence is 400 by second. The zero delay corresponds to the maximal overlap with a visibility of *v_a_* = 76.9% ± 0.9%, *v_b_* = 74.6% ± 0.8%, *v_c_* = 75.7% ± 0.8%, and *v_d_* = 74.6% ± 0.9% for the cases a, b, c, and d respectively. These non ideal values are due to the higher order emission contribution and to the imperfect photon spacial and spectral modes matching.

By using the MDI-EW method, we have experimentally tested the entanglement for a set of Werner two qubit mixed states by scanning the singlet weight *p* in the range of *p* ∈ {0, 0.95}. The referee send six different quantum inputs states to Alice and to Bob. In MDI-EW method, during the first phase, the referee estimate the visibilities *v_A_* and *v_B_* of the interference of Alice's and Bob's Bell analyzers respectively for different inputs quantum states sent by him. The referee arbitrarily selects a certain subset (which depends on the degree of the desired precision of the estimation) of Alice's results and the corresponding input states and independently another subset of Bob's results and corresponding inputs states. Our results show that these visibilities are independent of the inputs states *τ_s_* and *ω_t_* with the value 74%.

Due to the Alice 's and Bob's interference imperfections, the joint probabilities *P*(1, 1|*ω_s_*, *τ_t_*) becomes


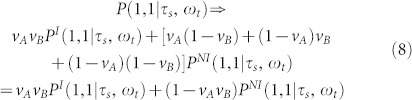
where *P^I^*(1, 1|*τ_s_*, *ω_t_*) and *P^NI^*(1, 1|*τ_s_*, *ω_t_*) are the probabilities for successful projection when input photons are interfering and non-interfering respectively. In the second phase, the referee uses the remaining set of the data to evaluate the twelve joint probabilities *P^exp^*(1, 1|*τ_s_*, *ω_t_*). Due to the independence of the visibilities *v_A_* and *v_B_* on its states |*τ_s_*〉 and |*ω_t_*〉 and therefore he can approximate the probability *P^NI^*(1, 1|*τ_s_*, *ω_t_*) = 1/4. Then he calculate the twelve experimental probabilities *P^exp^*^,*I*^(1, 1|*τ_s_*, *ω_t_*).

We have obtained *I*(*p* = 0.95) = −0.459 ± 0.065 and *I*(*p* = 0) = 0.250 ± 0.052 respectively. Several different values of the singlet weight *p* have been tested in the experiment. Figure 4 shows the experimental values of I(p) as function of *p*. The error bars are corresponding to the propagated Poissonian counting statistics. The obtained results shows a very good agreement with theoretical values. At the border between entangled and separable states (For the value of *p* = 0.356) we obtain *I* = −0.001 ± 0.041. All the multi-fold coincidences from the 10 counting detectors were recorded for the 12 combinations of the referee's settings for the quantum states sent to Alice and Bob, each setting being measured for one hour and half. The average six-photon coincidence is 23 by 10 seconds.

While editing this paper, we have learned that a related work has just been published^24^. Here, we have experimentally MDI detected the entanglement for very important class of mixed states, the so-called Werner states while they have detected the two qubit entanglement for another mixed states and different witness decomposition.

We have detected entanglement for mixed two qubit states in a measurement device independent way. This entanglement detection is based on the witness method. Our experiment can also be viewed as a demonstration of trust-free entanglement detection. It is also the realization of Buscemi game with quantum input data. These methods and techniques open the door for other applications i.e. the MDI determination of properties of quantum systems, fundamental tests of quantum mechanics, and the realization of quantum communication and cryptographic protocols. We believe that the results reported here will contribute to deeper understanding of foundations of quantum mechanics.

## Author Contributions

M.N., S.M, and E.A. carried out the experiment. N.M. analyzed the data. All authors discussed the results and wrote the manuscript. M.B. supervised the project.

## Figures and Tables

**Figure 1:**
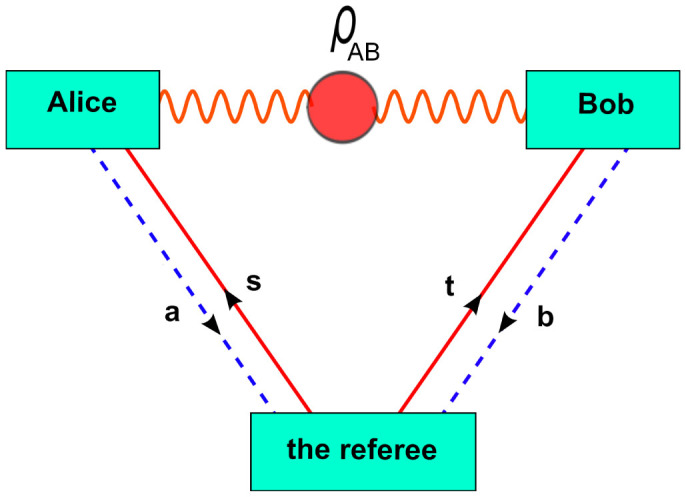
Quantum game Scheme. Two separated parties, Alice and Bob, receive quantum states *τ_s_* and *ω_t_* for Alice and Bob from a referee and must output values *a* and *b*, respectively. Alice and Bob are allowed to share some quantum state *ρ_AB_*. They can communicate before to agree on a pre-established strategy but they are not allowed to communicate during the game. The payoff of this game is characterized by the conditional probability distribution *P*(*a*, *b*|*τ_s_*, *ω_t_*).

**Figure 2:**
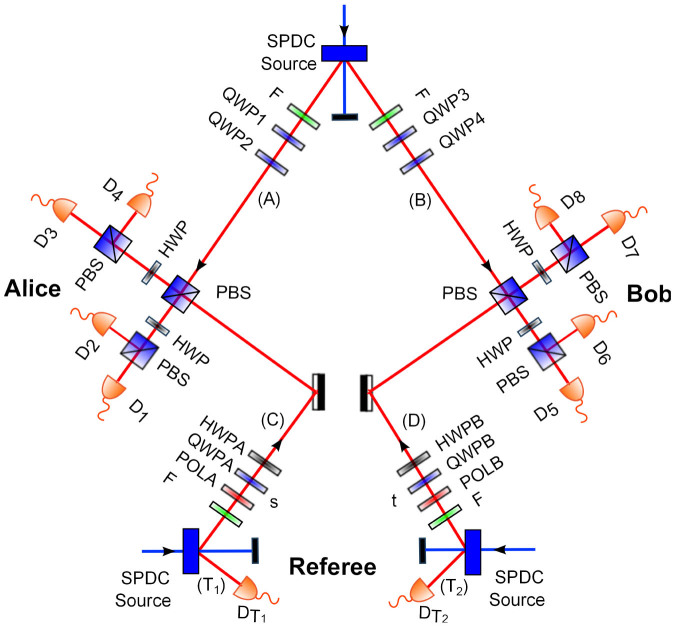
Experimental setup for the preparation of two qubit photon polarization mixed state, two single photon polarization states, and Bell Analyzers. The experimental Werner state preparation setup consist of photon pairs generation by spontaneous parametric down conversion (SPDC). These photon pairs are emitted by pumping nonlinear BBO with UV pulses. The four different Bell states are randomly prepared by rotating Quarter wave plates (QWP). The setup for single photon states consist of combinations of polarizer (POLA, POLB), hlaf wave plates (HWPA, HWPB), and QWP (QWPA, QWPB). Then the photons are brought to local Bell analyzers. The experimental setup for Bell analyzers consist of polarization beam splitter (PBS) and half waves plates (HWP) oriented at 22.5°. All measurements in the two output modes of Alice's and Bob's analyzers are performed with polarization analysis components. All the photons are coupled to single mode fibers (SMF) passing a narrowband filters, and they are detected by Si avalanche photodiodes *D_i_*(*i* = 1, …8, *T*_1_, *T*_2_).

**Figure 3:**
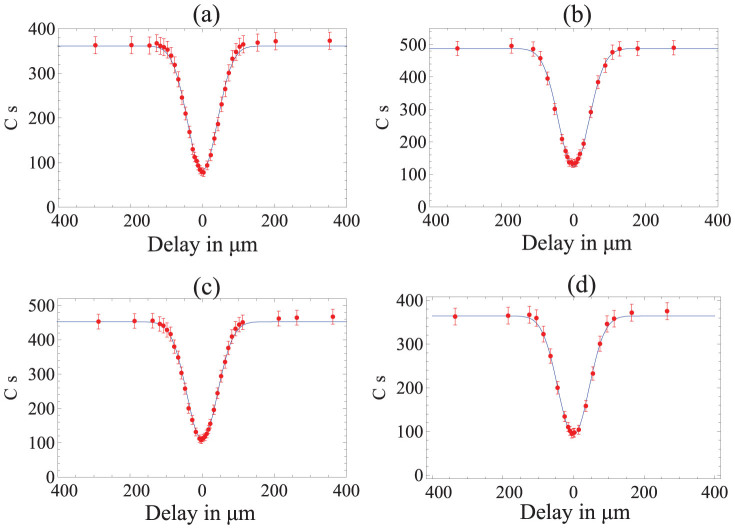
Four-fold coincidences for the Bell analyzers for Alice and Bob. The four-fold coincidence between the detectors versus the delay path between the photons A and C and B and D are shown. Sub-figures (a) and (c) show the four-fold coincidence for |*H*〉 and |*V*〉 input quantum states in mode C at Alice, singlet 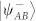 in modes A and B, and trigger photon in the mode *T*_1_. Sub-figures (b) and (d) show the four-fold coincidence for |*H*〉 and |*V*〉 input quantum states in mode at Bob, singlet 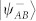 in modes A and B, and trigger photon in the mode *T*_2_.

**Figure 4:**
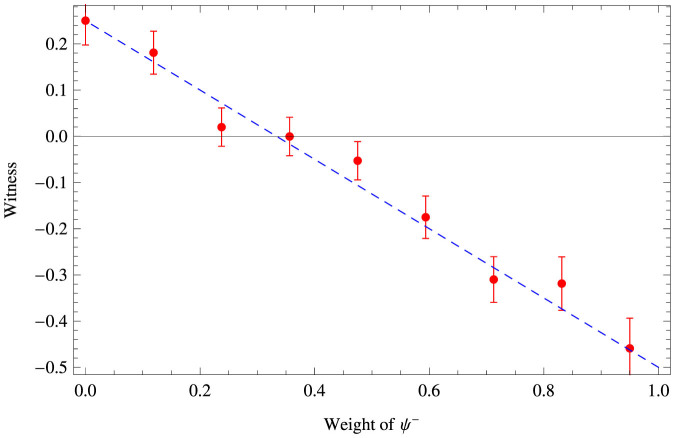
MID-EW results. Experimental results for the MDI-EW for two-qubit mixed Werner states for several different values of the singlet weight *p* ∈ {0, 0.95}. The line corresponds to the theoretical value.

**Table 1:** Settings for QWP for the preparation of Werner states

States/Settings	QWP1	QWP2	QWP3	QWP4
|*ψ*^−^〉	0°	90°	0°	90°
|*ψ*^+^〉	0°	90°	0°	0°
|*ϕ*^+^〉	0°	90°	45°	45°
|*ϕ*^−^〉	0°	0°	45°	45°

**Table 2:** Settings for Alice's and Bob's input quantum states: Polarizer (POLA and POLB), HWP (HWPA and HWPB), and QWP (QWPA and QWPB)

States/Settings	Polarizer	HWP	QWP
|*H*〉	0°	0°	0°
|*V*〉	0°	45°	0°
|*D*〉	0°	22.5°	0°
| 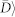	0°	−22.5°	0°
|*L*〉	0°	0°	45°
|*R*〉	0°	0°	−45°
